# Post-traumatic stress symptoms and burnout in healthcare professionals working in neonatal intensive care units: Results from the STRONG study

**DOI:** 10.3389/fpsyt.2023.1050236

**Published:** 2023-02-03

**Authors:** C. Ravaldi, L. Mosconi, L. Mannetti, M. Checconi, R. Bonaiuti, V. Ricca, F. Mosca, C. Dani, A. Vannacci

**Affiliations:** ^1^CiaoLapo Foundation for Perinatal Health, Prato, Italy; ^2^PeaRL – Perinatal Research Laboratory, Department of Neurosciences, Psychology, Drug Research and Child Health, University of Florence, Florence, Italy; ^3^Department of Experimental Medicine, University of Perugia, Perugia, Italy; ^4^Department of Health Sciences, Psychiatry Unit, Careggi General Hospital, University of Florence, Florence, Italy; ^5^Department of Pediatrics, Fondazione IRCCS Cà Granda Ospedale Maggiore Policlinico, Milan, Italy; ^6^Italian Society of Neonatology (SIN), Milan, Italy

**Keywords:** NICU, neonatal death, burnout, PTSD, healthcare professionals

## Abstract

**Background:**

Newborns’ deaths and life-threatening conditions represent extremely stressful events for parents and professionals working in NICUs, facilitating the onset of secondary traumatic stress symptoms. The STRONG study aims to better understand the psychological impact on Italian NICUs staff of bereavement care.

**Methods:**

The STRONG (STress afteR lOss in NeonatoloGy) study is a cross-sectional study based on a web survey consisted of four sections: sociodemographic, CommuniCARE-Newborn questionnaire, the Maslach Burnout Inventory and the Impact of Event Scale-Revised.

**Results:**

227 NICU workers (42.7% nurses, 23.3% midwives, 22.2% physicians, 11.8% other HCPs) answered the survey. The hardest tasks were “communicating baby’s death” and “informing on autopsy results”; 44.7% of HCPs did not receive formal training in communicating bad news, 44.2% ‘learned from the field’ by watching other colleagues; 41.2% declared that they do not have any communication strategy. More than 90% of professionals thought that training on bereavement care is necessary. The majority of HCPs showed some degree of post-traumatic stress symptoms: 34% medium and 35.3% severe. Professionals with training in bereavement care and/or in communication had less probability to develop stress symptoms. A multivariate analysis showed that higher levels of burnout were associated with 4 or more monthly losses and medium or severe stress symptoms. Having a well-defined communication strategy for breaking bad news was independently associated with a better personal accomplishment.

**Conclusion:**

Dealing with newborns’ deaths is a highly stressful task; professionals should receive proper support such as debriefing, psychological support and training in order to prevent post-traumatic stress symptoms and reduce professional burnout.

## 1. Introduction

Complications due to preterm birth are responsible for around 1 million of the world’s annual neonatal deaths (35% of the total) and are the second most common cause of death after pneumonia in children under 5 years old (1). Data from Italy shows that the average mortality in preterm infants is 15%, ranging from 84.3% in newborns less than 24 weeks of gestational age, to 4.5% in newborns more than 31 weeks of gestational age (2).

Health care providers (HCPs) who work in neonatal intensive care units (NICU) face neonatal death multiple times during their career. They routinely work with patients with life-threatening conditions, and this represents a source of secondary traumatization due to the stress derived from helping or wanting to help a suffering person. Stress is a physiological response to any internal or external condition perceived as a threat by an organism. Sometimes, the exposure to stressful events, in particular chronic ones, has the potential to promote the onset of stress disorders. This type of experience in the healthcare field has some similarities with the symptoms of Post-Traumatic Stress Disorder (PTSD), for instance: avoidance, intrusive imagery, distressing emotions and functional impairment (3). This burden could be aggravated by moral issues such as End-of-Life decisions, including many complex decisions about the baby and his family (4). In such difficult situations, HCPs could disagree about the most appropriate choice and sometimes feel forced to provide care they believe not to be in the interest of the baby resulting in moral distress (“doing things to the baby instead of for the baby”) (5, 6). Switching from a curative model of care to palliative care may be very hard; moreover, the grieving process over the death of a baby is usually more complicated than that over the death of an adult patient and is characterized by a deep feeling of failure (7). Moral issues may lead to burnout syndrome (8, 9) and impact on patient care (10). Burnout syndrome is defined in ICD-11 as an occupational phenomenon and not a medical condition. It is conceptualized as “resulting from chronic workplace stress that has not been successfully managed” and is characterized by feelings of exhaustion, reduced professional efficacy and increased mental distance from one’s job. This syndrome is not taken into account in DSM-5 which, however, has a distinct category for stress disorders unlike the fourth edition of the manual (11–13).

Although there is a high heterogeneity in research papers focused on support interventions for HCPs working in NICU, all studies agree they should be directed to both individuals and organizations (14), adapting healthcare systems to patients’ and HCPs’ features, rather than vice versa (15). Staff members should be supported with proper training in bereavement care based on evidence-based practices and tailored on their needs (16). Formal and informal systems to support HCPs such as debriefing, peer group support, professional counseling should be put in place and be available for everyone directly or indirectly involved with trauma and sudden death (17–22). In particular, staff concerns about moral issues should be taken into account by the hospital, which should offer support for ethical decision making (23–25). Moreover, as shown in the literature, individual levels of resilience and one’s ability to tolerate uncertainty are factors that play an important role in the onset of burnout symptoms (26). So, they should be taken into account to design proper interventions to support HCPs.

Communication training, with the aim to improve the ability of HCPs to empathize and understand psychological needs of patients, has the power to positively influence the risk of developing burnout symptoms among staff members working in palliative care fields (27–29) such as NICU. However, neonatologists often receive an inadequate education in communication skills (30) and fail to meet the families’ needs (31, 32). Another useful instrument to enhance patient care and decrease staff’s level of stress could be trauma-informed care. This approach was implemented in the substance abuse field at first and later applied in other medicine areas such as in NICU (33, 34). As mentioned before, HCPs may suffer secondary traumatization: trauma-informed care allows to create social connectedness between staff and parents leading to a decreased stress level (34) which is known to be linked to burnout syndrome.

We have previously shown that burnout in midwives, measured especially in the emotional exhaustion and depersonalization subscales of the Maslach Burnout Inventory, significantly correlated with the psychological impact of stressful events (measured by IES-R), such as stillbirth (35). An important protective factor is professional training on bereavement care (35) which is generally inadequate (36) as mentioned before for neonatologists. To better understand the psychological impact of bereavement care in Italian NICUs’ staff, with particular attention to professional burnout symptoms, we designed the STRONG study (STress afteR lOss in NeonatoloGy).

## 2. Materials and methods

A cross-sectional study design was used. The CommuniCARE-Newborn questionnaire was developed by CR and AV and uploaded as an online survey using the Surveymonkey platform.^1^ The survey was distributed by CiaoLapo Foundation, an Italian organization for the promotion of perinatal health, in collaboration with several Italian hospitals and the Italian Society of Neonatology (Società Italiana di Neonatologia, SIN). The study was approved by Florence University ethics committee (Prot. n. 0233044/2020). All data were collected between September 2020 and December 2021, and analyzed anonymously. Participants were considered eligible to complete the survey if working in NICU. The sample was made of nurses, midwives, physicians and other HCPs (psychologists, psychotherapists and rehabilitation therapists). Consent was provided at the beginning of the survey once participants had read participant information and met eligibility criteria.

Each subject was asked to complete the survey consisted of questions across several key areas including: (1) Sociodemographic information; (2) Section A – CommuniCARE-Newborn; (3) Section B – Maslach Burnout Inventory; (4) Section C – Impact of Event Scale Revised.

The CommuniCARE-Newborn questionnaire was developed by the authors in order to explore knowledge and feelings of HCPs towards neonatal death management in NICU. In particular, this questionnaire includes closed and open-ended questions focused on: tasks deemed more difficult to face by HCPs, training about communication and bereavement care, HCPs’ perception about their competency in perinatal loss area, HCPs’ emotional and clinical experience about perinatal loss. The Maslach Burnout Inventory – Human Services Survey for Medical Personnel (MBI-HSS MP) is based on the original MBI and is specifically used to assess the level of professional burnout in HCPs. It is a 22-item survey and each item reflects the professionals’ feelings and attitudes toward their work and their patients. MBI-HSS MP covers three areas: Emotional Exhaustion (EE) which includes statements about physical and mental health status, Depersonalisation (DP) where an impaired and distorted perception of oneself and others is investigated, sense of low Personal Accomplishment (PA) which explores the tendency to negatively evaluate the worth of one’s work. Each subscale includes multiple questions with frequency rating choices from Never to Every day (Likert-type scale from 0 to 6). The Italian version of MBI-HSS MP was licensed by Mind Garden INC to CR for the realization of this study.

The Impact of Event Scale Revised (IES-R) is a 22-item self-report measure to assess subjective distress caused by traumatic events. Respondents were asked to indicate how much they were distressed or bothered by each item listed with reference to events of neonatal death they had taken care of, on a Likert-type scale ranging from 0 (Not at all) to 4 (Extremely). The IES-R was originally designed to be used in recent traumatic events, nevertheless over the years it has been widely used to address PTSD symptoms of remote events (33). For the purposes of this research, we used IES-R to evaluate symptoms of post-traumatic stress, not as a diagnostic tool. The questionnaire comprises three main post-traumatic symptoms subscales (intrusion, avoidance, and hyperarousal). The outcome of each subscale was analyzed as a continuous measure (mean value). The total score was calculated as a continuous measure (sum) which ranged from 0 to 88; higher scores indicated more post-traumatic stress symptoms. Using quartiles distribution, IES-R was also classified as very low (<10), low (10,11,12,13,14,15,16,17,18,19,20), moderate (21,22,23,24,25,26,27,28,29,30) and high (>30). Although not diagnostic, high IES-R values suggest the presence of clinically relevant post-traumatic stress symptoms. The IES-R version used in this study is in the public domain.

### 2.1. Statistical analysis and data presentation

Survey responses were downloaded and extracted from the online survey tool Surveymonkey and imported into Excel for data management. Incomplete records were excluded and quantitative data were imported into Stata/BE 17.0 (StataCorp) for statistical analysis. Descriptive statistics were used to analyze quantitative data. Categorical data were reported as frequencies and percentages and compared using the chi-squared test, whereas continuous data were reported as mean values with standard deviations (SD) or as median [quartiles] and compared using t-test or Kruskal Wallis and Mann Whitney test. All results were considered to be statistically significant at *p* < 0.05.

A linear regression was performed using IES-R score as independent variable and each MBI subscale score as dependent ones using Pearson product–moment correlation coefficient; a multivariate analysis (ordered logistic regression) was performed to evaluate the association between MBI subscales and several variables. The following parameters were used as covariates of three ordered logistic models having MBI subscale scores as dependent variables: age, number of work years, number of loss assisted, history of training in communication, history of training in perinatal loss management, presence of structured communication strategies. The analysis provided the OR [95% CI] for being in the highest third of each MBI subscale.

Statistics were performed with Stata/BR 17.0 (StataCorp) whereas the map of respondents and the map of scores at MBI subscales across Italy were plotted using Tableau Desktop 2022.1 (Tableau Software, LLC).

## 3. Results

### 3.1. Sample characteristics

**Figure 1:**
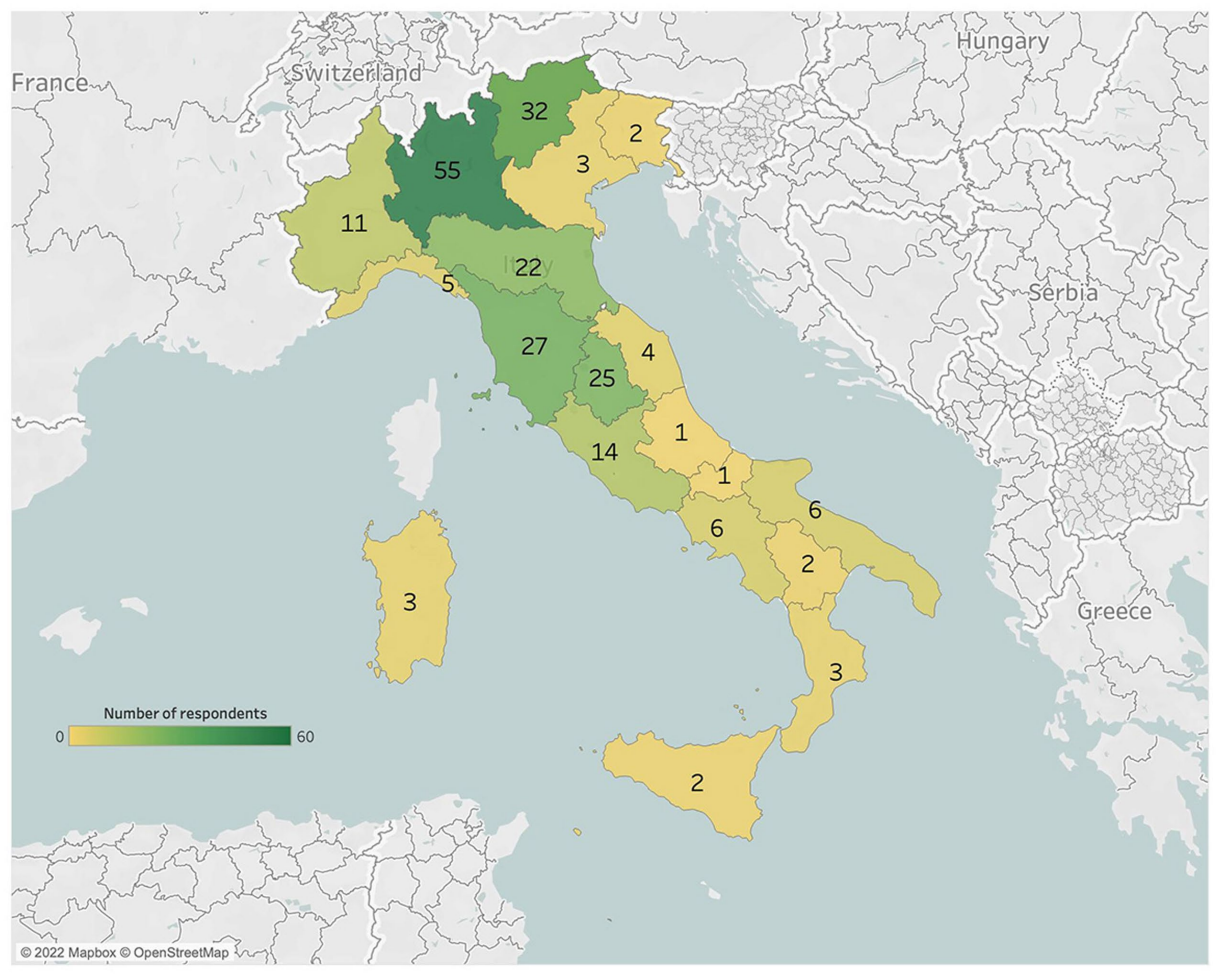
The map shows the geographical distribution of HCPs which answered the survey.

A total of 227 HCPs (9.7% M and 90.3% F) took part in this study. The responders came from all over Italy, but the majority of them worked in the North (133, 58.5%) and Center (71, 31.2%; Figure 1). The sample was made of a majority of nurses (Table 1). Significant differences were present between groups regarding age, number of monthly losses assisted and years of work (Table 1).

**Table 1:** Characteristics of the sample.

	Job											
	Physician		Midwife		Nurse		Other		Total		χ^2^	*p*
	No.	%	No.	%	No.	%	No.	%	No.	%		
Age classes												
≤30	8	14.5%	33	62.3%	24	24.7%	11	50.0%	76	33.5%	45.79	<0.001
31–50	18	32.7%	18	34.0%	36	37.1%	6	27.3%	78	34.4%		
>50	29	52.7%	2	3.8%	37	38.1%	5	22.7%	73	32.2%		
Monthly losses												
<1	11	21.2%	31	75.6%	47	52.2%	11	68.8%	100	50.3%	38.52	<0.001
1–2	18	34.6%	7	17.1%	26	28.9%	5	31.2%	56	28.1%		
3–4	10	19.2%	2	4.9%	9	10.0%	0	0.0%	21	10.6%		
>4	13	25.0%	1	2.4%	8	8.9%	0	0.0%	22	11.1%		
Years of work												
<5	8	14.5%	33	62.3%	24	24.7%	11	50.0%	76	33.5%	45.79	<0.001
5–15	18	32.7%	18	34.0%	36	37.1%	6	27.3%	78	34.4%		
>15	29	52.7%	2	3.8%	37	38.1%	5	22.7%	73	32.2%		
Total	**55**		**53**		**97**		**22**		**227**			

“Other” category included psychologists, physiotherapists and rehabilitation therapists.

**Figure 2:**
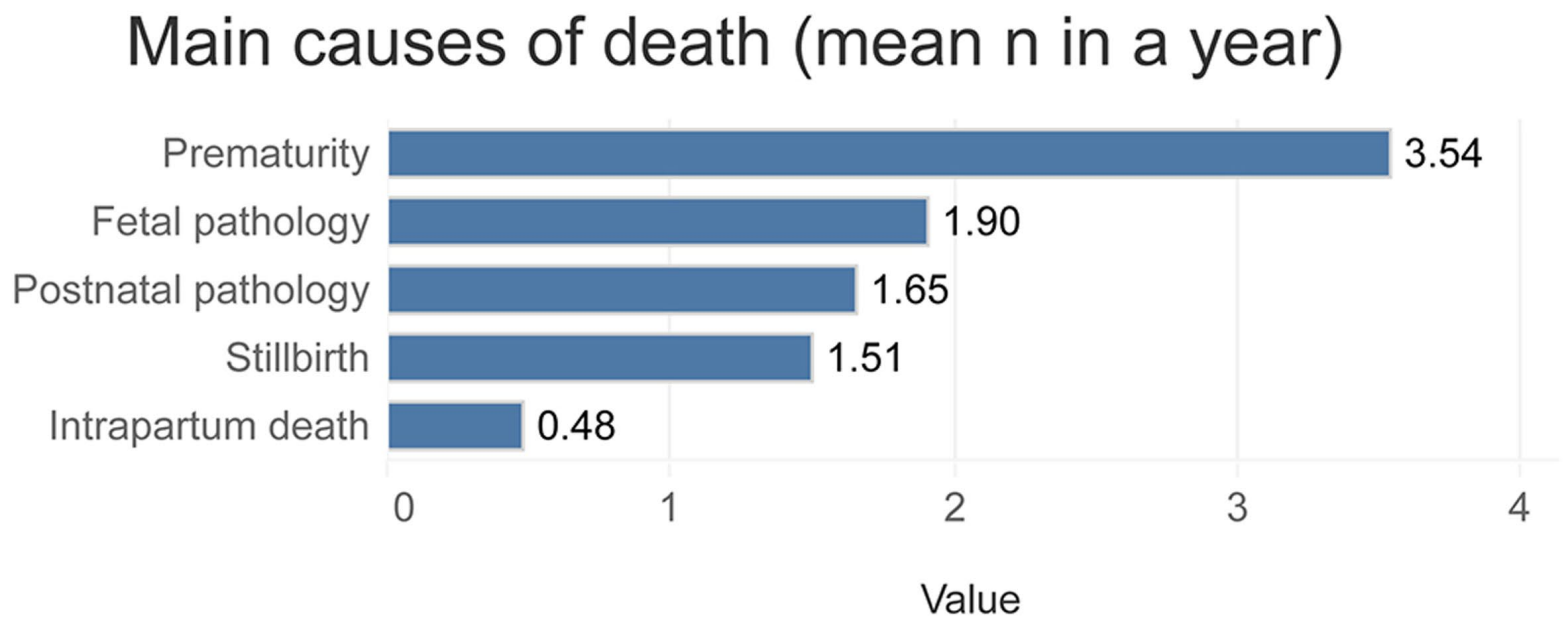
Main causes of neonatal death faced by HCPs.

Prematurity was the most frequent cause of death faced by professionals in our sample (mean 3.5 deaths per year, SD 4.8), followed by fetal pathology (a mean of 1.9 deaths per year, SD 2.5; Figure 2). In our sample, almost all NICUs allowed parents to stay during the hospitalization (95.2%), immediately before the baby’s death (94.6%) and after the baby’s death (96.8%).

### 3.2. Breaking bad news

**Figure 3:**
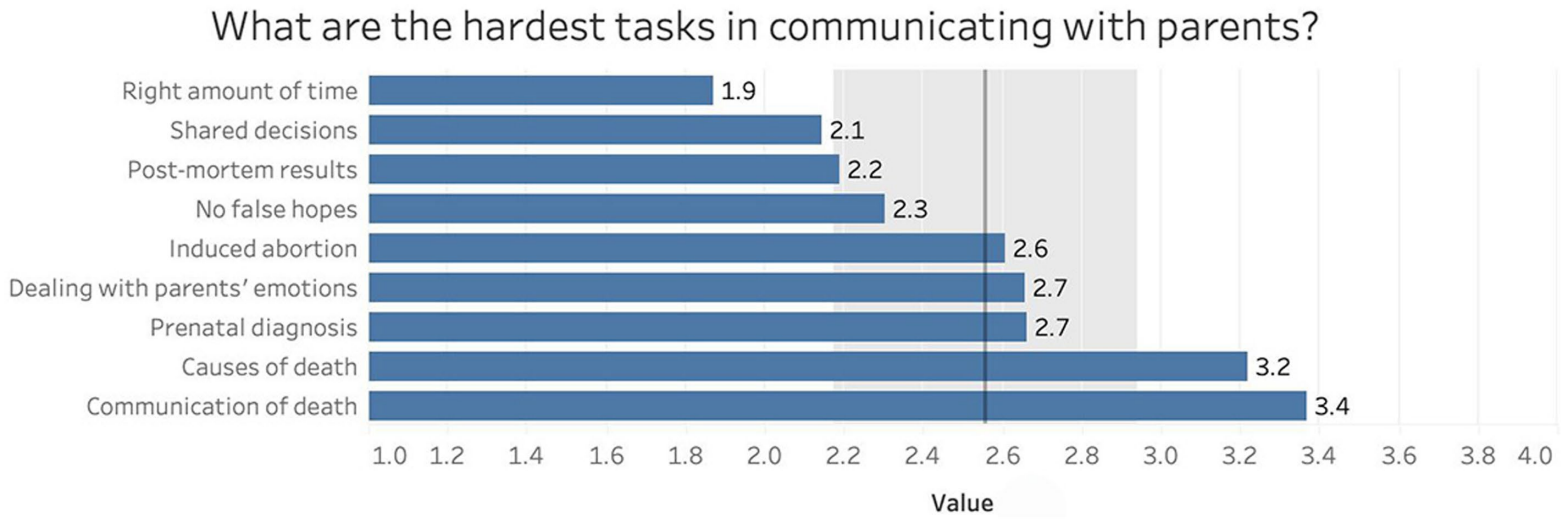
The hardest tasks for HCPs in communicating with parents.

Figure 3 reports communication tasks with parents considered most difficult by HCPs. Tasks were graded using a Likert scale (from 0 “not at all” to 4 “very much”). Communication of the cause of death (mean 3.2, SD 1.0) and death itself (mean 3.4, SD 0.9) represented the hardest duties, while finding the right amount of time for communication was considered the easiest (mean 1.9, SD 1.1).

Table 2 shows HCPs’ most frequent feelings during the meeting between parents and their deceased baby. The results are divided between physicians, midwives and nurses and underline that impotence (65.2%) and compassion (63.1%) were the most common emotions.

**Table 2:** Emotions most frequently reported when assisting parents’ meeting with their dead baby.

	Job											
	Physician		Midwife		Nurse		Other		Total		χ^2^	*p*
	No.	%	No.	%	No.	%	No.	%	No.	%		
Anxiety	0	0.0%	5	13.5%	12	14.1%	1	7.1%	18	9.6%	8.14	0.04
Fear	2	3.9%	6	16.2%	3	3.5%	4	28.6%	15	8.0%	14.86	<0.01
Uneasiness	9	17.6%	16	43.2%	26	30.6%	2	14.3%	53	28.3%	8.49	0.03
Impotence	29	56.9%	28	75.7%	56	65.9%	9	64.3%	122	65.2%	3.37	0.33
Compassion	34	66.7%	26	70.3%	49	57.6%	9	64.3%	118	63.1%	2.19	0.53
Endearment	27	52.9%	15	40.5%	43	50.6%	11	78.6%	96	51.3%	5.95	0.11
Solidarity	29	56.9%	17	45.9%	42	49.4%	7	50.0%	95	50.8%	1.16	0.76
Others	5	9.8%	7	18.9%	12	14.1%	1	7.1%	25	13.4%	2.05	0.56

“Others” category includes: sadness, pain, anger and love.

### 3.3. Training in bereavement care: How it influences HCPs practice

56.3% of HCPs had formal training in communication and 62.5% of HCPs had formal training in perinatal loss care. 44.2% of the sample learned communication skills from other senior colleagues (Table 3). More than 90% of professionals thought that training on perinatal loss and bereavement care is necessary or indispensable to take care of babies and families during the hospitalization, just before death and after. Regarding communication strategy, 82 professionals (41.2%) reported having no strategy at all, 96 of them (48.2%) have some kind of unstructured strategy that changes every time, and only 21 (10.5%) reported having a structured specific communication strategy for bad news.

**Table 3:** Previous training in communication and in perinatal loss.

	Communication		Perinatal loss	
	No.	%	No.	%
No training at all	58	29.1%	53	28.0%
Formal university training (before graduation)	38	19.1%	30	15.9%
Postgraduate training	74	37.2%	88	46.6%
Learned from the field	88	44.2%	68	36.0%

**Figure 4:**
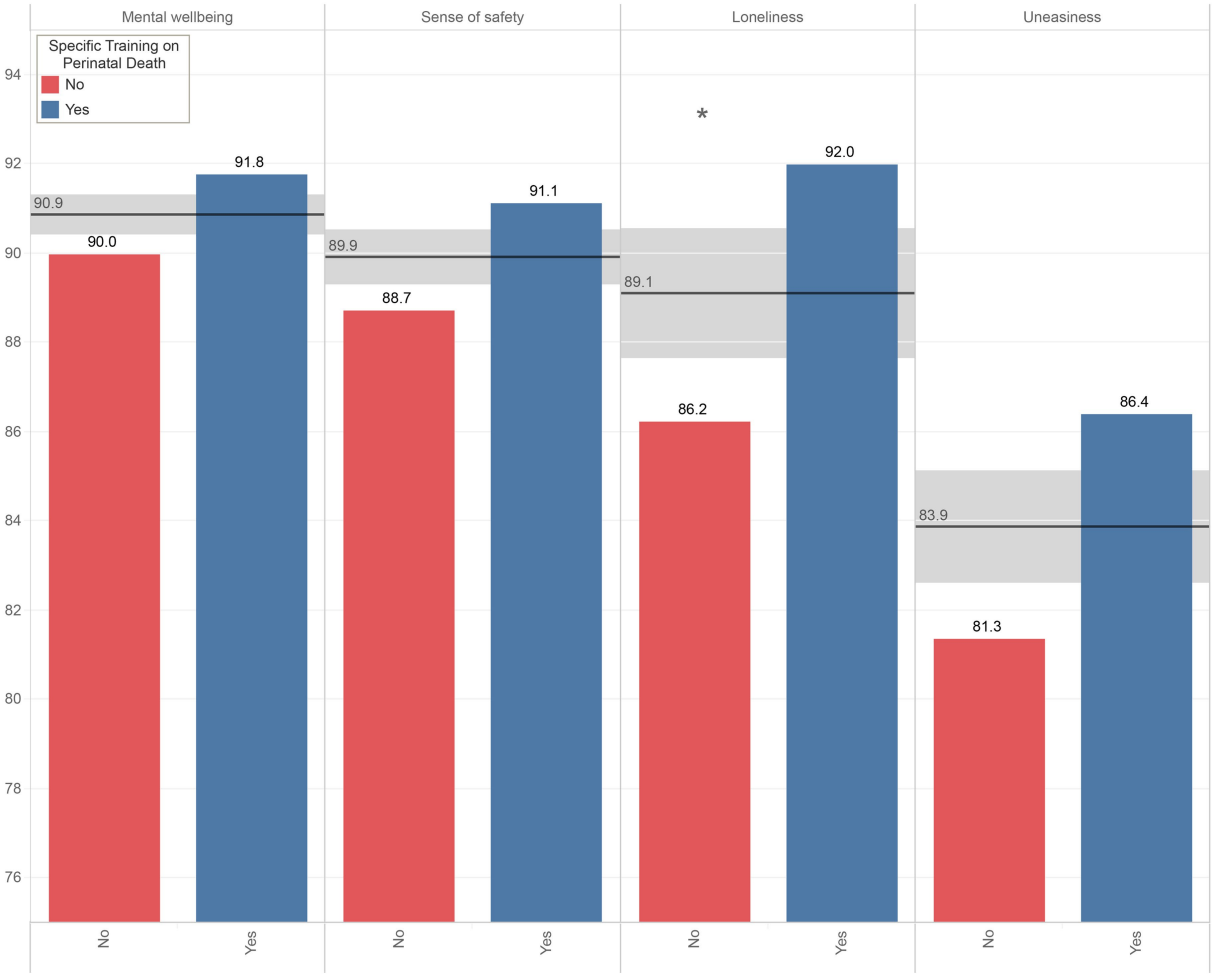
Percentages of HCPs who considered the relationship between physician and patient to be important when a neonatal death occurred in the areas of patients’ mental wellbeing, sense of safety, feeling of loneliness and feeling of uneasiness.

Figure 4 shows the percentages of HCPs who considered the relationship between physician and patient to be important when a neonatal death occurred. The areas examined deal with the effects of the relationship on patients’ mental wellbeing, sense of safety, feeling of loneliness and feeling of uneasiness. Results are divided between professionals who received specific training in bereavement care and those who did not. HCPs who received a specific training reported significantly higher scores in valuing the importance of doctor-patient relationship in reducing patients’ loneliness.

**Figure 5:**
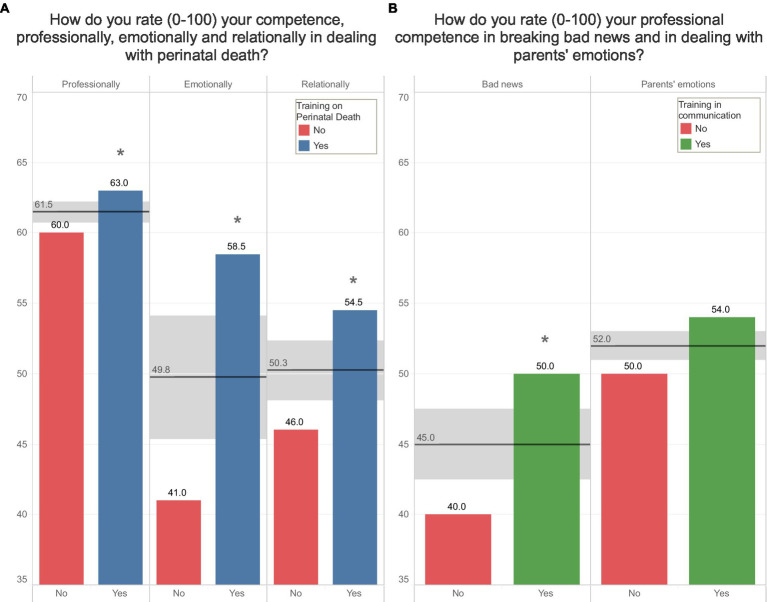
Panel **(A)** shows the differences between HCPs having a specific training in bereavement care and those who do not on how they rate their competencies (from 0 to 100) in bereavement care. Panel **(B)** shows the differences between HCPs having a specific training in communication and those who do not on how they rate their competencies (from 0 to 100) in breaking bad news and dealing with parents’ emotions.

Figure 5 shows how HCPs rate their competency (from 0 to 100) regarding bereavement care. Those who had received a specific training on perinatal death (panel A) or communication (panel B) showed more self-confidence in dealing with loss and breaking bad news than other colleagues. Most difficult tasks were considered facing parents’ emotions, facing their own emotions and breaking bad news.

Table 4 shows emotional and psychological support figures chosen by HCPs for debriefing. Colleagues were the main resource (89.3%) followed by relatives and friends (43.3%). Having received a specific training on perinatal death influenced HCPs choice regarding the type of support preferred: trained professionals referred in fact more frequently to private psychologists (*p* < 0.05).

**Table 4:** Figures most commonly used for debriefing.

	Perinatal death training							
	No		Yes		Total		χ^2^	*p*
	No.	%	No.	%	No.	%		
No one	6	7.6%	3	2.8%	9	4.8%	2.31	0.12
Relatives/friends	37	46.8%	44	40.7%	81	43.3%	0.69	0.40
Colleagues	67	84.8%	100	92.6%	167	89.3%	2.89	0.08
Hospital psychologist	5	6.3%	15	13.9%	20	10.7%	2.73	0.09
Private psychologist	5	6.3%	17	15.7%	22	11.8%	3.89	<0.05

### 3.4. The burden of working in NICU: HCPs’ mental health

Table 5 shows how HCPs answered to IES-R: the majority of them had medium or high levels of PTSD-like symptoms (34.0% medium level and 35.3% high level). Although not statistically significant, midwives seemed to be the most vulnerable category between HCPs: 44.1% of them reported high levels of PTSD-like symptoms.

**Table 5:** Level of PTSD-like symptoms (measured by IES-R) according to different professions.

	Job									
Stress symptoms	Physician		Midwife		Nurse		Total		χ^2^	*p*
	No.	%	No.	%	No.	%	No.	%		
Low	15	33.3%	7	20.6%	26	33.8%	48	30.8%	2.593	0.628
Medium	16	35.6%	12	35.3%	25	32.5%	53	34.0%		
High	14	31.1%	15	44.1%	26	33.8%	55	35.3%		
Total	45	100.0%	34	100.0%	77	100.0%	156	100.0%		

Prior communication and/or perinatal death training was shown to influence the severity of PTSD-like symptoms. Professionals who received communication training were less likely to develop high levels of symptoms at the subscales of avoidance (21.2% vs. 43.6%, χ^2^ = 9.9, *p* = 0.007) and hyperarousal (27.5% vs. 39.2%, χ^2^ = 11.9, *p* = 0.003). Moreover, having perinatal death training led to a lower probability of developing avoidance symptoms (IES-R avoidance mean score: 0.9 SD 0.6 vs. 1.2 SD 0.7; *p* < 0.05).

**Figure 6:**
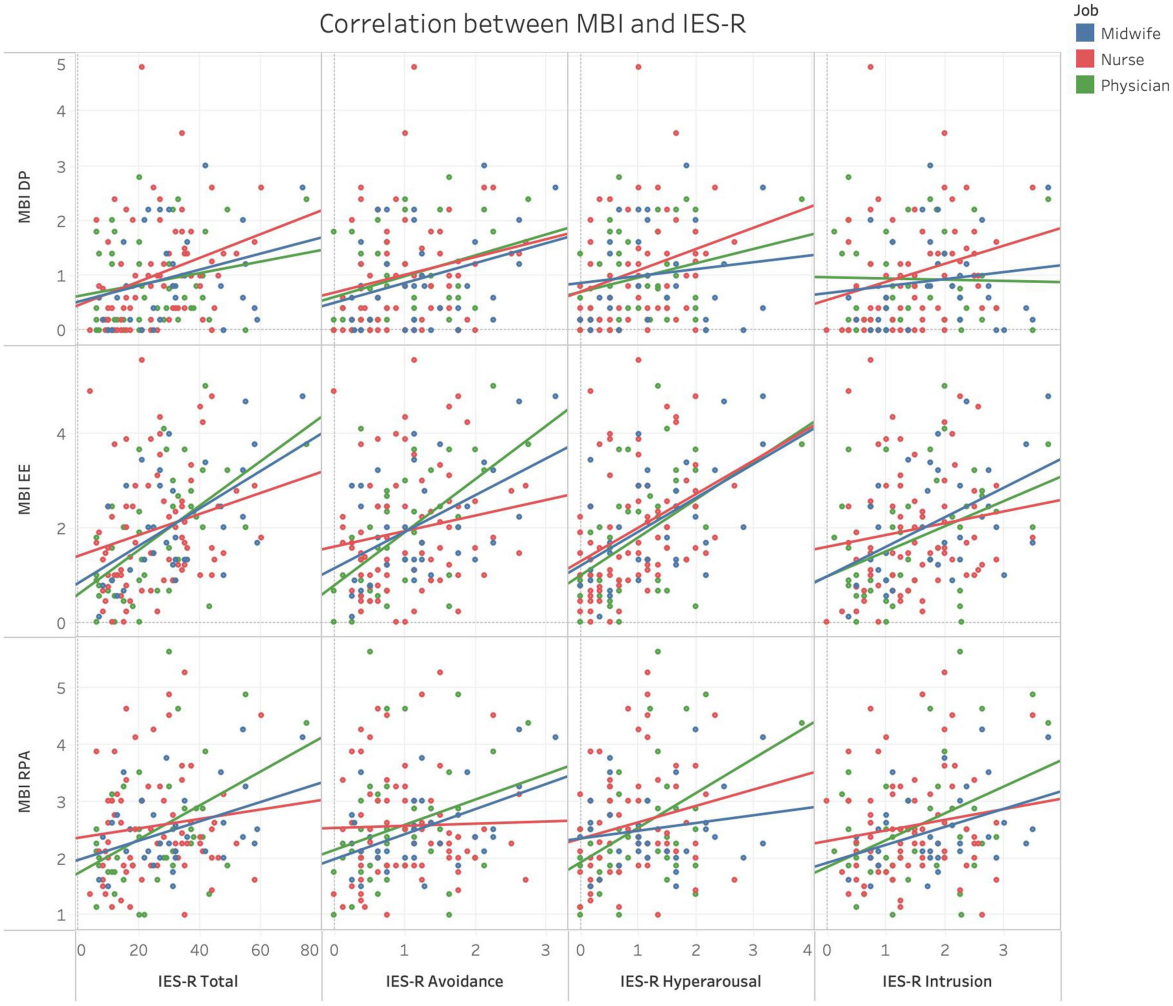
Scatter plots showing correlations between MBI subscales scores and IES-R total and subscales scores in different HCPs categories.

All scores of MBI subscales and IES-R total and subscale scores were significantly correlated (Supplementary Table 1); Figure 6 graphically depicts different strengths of association in different HCPs categories.

**Figure 7:**
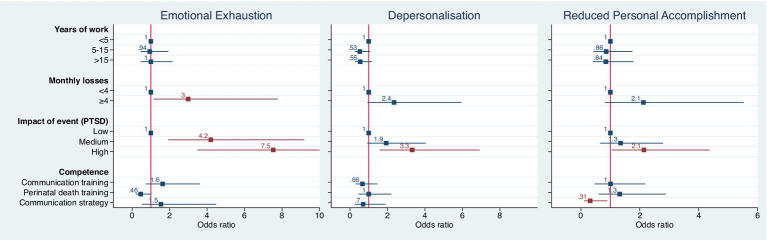
Results of a multivariate analysis showing associations between MBI subscales with several independent factors; red color *p* < 0.05.

In a multivariate analysis (Figure 7) MBI subscales were found to be associated with several independent factors, in particular, assisting 4 or more monthly losses and scoring medium or high levels of PTSD-like symptoms increased the risk of developing emotional exhaustion. The presence of high severity PTSD-like symptoms increased more than 3 times the risk of developing depersonalization and doubled the risk of developing a reduced personal accomplishment.

Instead, reporting to have a well-defined communication strategy was the only independent protective factor for the MBI reduced personal accomplishment subscale.

## 4. Discussion

### 4.1. Breaking bad news

Providing end of life care is very challenging for HCPs and can result in negative psychological responses such as feelings of frustration, guilt, burnout, moral distress, secondary traumatic stress, depressive and psychosomatic symptoms (37, 38). Staff working in intensive care units are more vulnerable than other HCPs to develop moral distress due to additional stressors they encounter: they routinely face ethical dilemmas and the threat of committing errors with serious consequences (39, 40). They have to deal with one traumatic event after another, leaving only a little time for recovery (41, 42).

Moreover, literature shows that HCPs often lack proper training in perinatal bereavement care, especially in breaking bad news (35, 43), making communication of the death of the baby even tougher. Our study confirms these findings: the hardest duties underlined by HCPs were death itself and communication of the cause of death.

To better understand the experience of HCPs during the encounter between parents and their deceased baby, we have explored their feelings: impotence and compassion were the two most common ones, and they were present in more than 60% of staff. We can speculate that a feeling of impotence could favor the onset of burnout symptoms leading the professional to a state of emotional exhaustion, and diminished satisfaction with their job (44–46).

### 4.2. Training in bereavement care

As mentioned before, neonatologists often receive inadequate formal communication training during the years they spend at university (30). The same situation was found in the sample here studied, with the majority of HCPs who actually received training in bereavement care did so only in specific courses after graduation. Furthermore, 44.2% of recruited participants learned communication skills only by observing other colleagues; their competences might therefore be incomplete as they never had the possibility to receive feedback and to practice in a structured educational environment (30). The consequences of this situation are quite evident since almost 90% of HCPs reported to have no strategy or an unstructured communication strategy, that changes every time, to breaking bad news.

Literature about patient-centered care underlines instead that empathy, respect and good communication are of paramount importance to establish a positive doctor-patient relationship (47). This essential achievement was valued more significantly by HCPs with specific training in bereavement care: greater knowledge leads the professionals to better understand the beneficial effects of a good relationship with their patients and improves their self-confidence in dealing with death and breaking bad news. It is possible that having a formal training helps HCPs to reflect more deeply on how the notions they have learned impact their clinical practice (48).

Although having training in perinatal loss care influenced self-confidence, both groups of HCPs rated poorly their competencies about breaking bad news, dealing with their own emotions and with parent’s emotions when the baby died. In a previous study on midwives, we found that lack of a specific education in bereavement care was perceived as the main obstacle to deal with stillbirth (36) so the knowledge of international guidelines about care of perinatal loss could represent an important achievement also for staff working in NICU improving assistance and coping in HCPs. The difficulties in breaking bad news that here we highlight in neonatal settings, are in line with what we observed in 2021 (35) on a sample of midwives working in obstetrics departments. Formal training and proper support, such as formal debriefing (49) where staff can express the feelings elicited by patient loss, could reduce the emotional impact of the experience, and help professionals caring for their other patients, given the short time to grieve.

### 4.3. Post-traumatic stress and burnout symptoms

It is known that staff working in NICU can easily face psychological difficulties. Results of STRONG investigation fully confirm this observation: almost 70% of HCPs here interviewed reported medium or high levels of PTSD-like symptoms. Nevertheless, to our knowledge this is the first study that explores the presence of post-traumatic stress symptoms related to newborn death in NICU. Due to the low percentage of male respondents, we cannot assess any correlation between gender and severity of post-traumatic stress or burnout symptoms. Literature shows an abundance of studies about this topic which should be investigated in NICU too (50, 51).

Despite the high number of studies exploring PTSD-like symptoms in parents of babies cared for in neonatal units, very few ones explored symptoms in bereaved parents and no such research was yet conducted on professionals working in NICU. While one study explored secondary traumatic stress in nurses and midwives working in maternity departments (52, 53), here we also included physicians and other staff, since bereaved parents often report the importance of being cared for by the whole staff before and after the death of the baby. Most recent guidelines on bereavement care also highlight the importance of training and debriefing for all staff members (54). Comparing STRONG results with the two studies conducted on secondary traumatic stress, we can observe that in our sample nurses and midwives reported higher level of post-traumatic stress symptoms: 66.3% of nurses reported medium or high level of PTSD-like symptoms vs. 50% (52), and 79.4% of midwives reported medium or high level of PTSD-like symptoms vs. 26.9% (53). We could hypothesize that the psychological burden of nurses and midwives was higher than that of physicians due to the closeness of these professionals to patients and families. Moreover, a study of McMillen underlines that the exclusion of nurses in end-of-life decisions could cause feelings such as anger and frustration and if they did not agree with such decisions, burnout symptoms could arise (55).

The samples are not of course completely comparable: first of all background are quite different, one study was performed in United States (52) and the other in French-speaking Switzerland (53), but most of all the instrument used was the Secondary Traumatic Stress Scale (STSS), a self-report questionnaire specifically designed for professional caregivers. In the STRONG study the focus was instead on the impact of a specific traumatic event (neonatal death) on professionals, with particular reference to the emotional consequences of the event. So, we decided to use the IES-R to explore the specific impact of babies’ death (and not of working in a NICU setting in general) on HCPs wellbeing, and we found that traumatic event related emotions can be very disturbing and if not managed they can eventually lead to burnout.

In fact, having a specific training in bereavement care or in communication influenced HCPs’ mental health both directly and indirectly, as PTSD-like symptoms as well as burnout symptoms were less severe. Likewise, the absence of a structured communication strategy was found to be linked to reduced “personal accomplishment”: again, knowledge represents a protective factor for professionals’ mental health. Having received a specific training on perinatal death also influenced HCPs choice regarding the type of support they seek: trained professionals referred in fact more frequently to private psychologists. This may be due to the fact that being formally trained in perinatal loss includes the knowledge of the great stress that such events exert on professionals, possibly leading to psychological distress, that is likely to need a specific intervention. Formal training in perinatal loss might also reduce the stigma towards needing a psychological intervention, so that trained professionals might be more likely to seek help when they need it, instead of hiding their feelings.

Our sample reported to care for a notable number of monthly losses, and higher numbers were associated with increasing levels of emotional exhaustion. This means that HCPs working in hospitals with a major workload and facing frequent losses will reasonably encounter more emotional difficulties and may be in turn less prepared to care for patients, creating a vicious circle. However, we did not find a correlation between burnout symptoms and years of work as shown in other studies (56). It is also well known that professionals’ burnout can have serious consequences on patient safety, as shown in a review published in 2016 (57), and on professionals’ quality of life by decreasing self-compassion (58).

Finally, more than 90% of HCPs from our sample thought that training on perinatal loss care was indispensable to provide proper care to families and babies. We believe that specific training and support should be the most important factors to improve HCPs’ mental health and clinical practice. A study conducted in the United States (49) showed the beneficial effects on stress levels of a formal debriefing program for staff working in NICU. Another research conducted in Italy, on a sample made predominantly of nurses, underlined a higher level of general health measured with SF-36 and a reduction in absenteeism after establishing a psychological support program for hospital workers (59).

## 5. Limitations

The aim of this study was to assess the impact of cumulative stressors on HCPs working in NICU. However, a critical point is the impossibility to clearly distinguish between PTSD symptoms and symptoms of acute stress due to the absence of specific information about the time since the event which elicited reported stress symptoms. For this reason, further research is needed to investigate this aspect, since a proper diagnosis is fundamental to design interventions tailored to HCPs’ needs.

Another limit is the low presence of male respondents which prevented any inference about gender role in determining pathological stress response. As mentioned before, this correlation is well documented in literature and should be studied in depth also in the field of NICUs.

## 6. Conclusion

Results from the STRONG study confirm that providing end of life care is very demanding for HCPs. Italian staff working in NICUs are particularly at risk of developing post-traumatic stress and burnout symptoms. A high score at the “emotional exhaustion” subscale of MBI was significantly correlated with the number of monthly losses and having a specific training in bereavement care was the only independent protective factor for both post-traumatic stress and burnout symptoms. However, only half of our sample had received a formal education in the field and the majority of them did so only after graduation. Although we are aware that other factors, not investigated here, could influence HCPs’ wellbeing and mental health, we can conclude that professional training is of paramount importance in promoting mental wellbeing, self-confidence and professional skills. We suggest that appropriate psychological support programs and scheduled debriefing sessions should be implemented for NICU staff; this could be particularly useful in reducing post-traumatic stress and burnout symptoms, improving care, patient safety and HCPs’ quality of life. Further research is needed to address the feasibility and actual effectiveness of such interventions.

### 6.1. Statement of significance

Newborns’ deaths and life-threatening conditions are very stressful events for parents and health care professionals (HCPs) working in neonatal intensive care units (NICUs). These situations represent a source of secondary traumatization, which is a peculiar condition of the helping professions, and has some similarities with Post-Traumatic Stress Disorder (PTSD). This burden can be further aggravated by moral issues such as End-of-Life decisions which are known to be linked to burnout syndrome that impacts on professionals’ life and patient care. Research has long been focused on mental well-being of families hospitalized in NICU and little data is available about the impact of bereavement care on HCPs. The STRONG study aims to better understand the psychological status of Italian NICUs staff and the role of specific training in bereavement care. Our results showed that almost half of our sample did not receive formal training in bereavement care. Most HCPs showed medium or severe degree of post-traumatic stress symptoms which were associated with burnout syndrome. Finally, professionals with training in bereavement care and/or in communication had less probability of developing post-traumatic stress symptoms. This paper highlights the impact of bereavement care on HCPs and the importance of specific training as a protective factor.

## Data availability statement

The raw data supporting the conclusions of this article will be made available by the authors, without undue reservation.

## Ethics statement

The studies involving human participants were reviewed and approved by Human Research ethical approval to conduct the survey was received from Florence University Ethics Committee (Prot. n. 0233044). Each participant gave their explicit consent in an online form before enrolment. Written informed consent for participation was not required for this study in accordance with the national legislation and the institutional requirements.

## Author contributions

CR and AV led this research including proposal write up and designed the instrument. CR, AV, LMa, MC, and RB collected the data. AV and RB analyzed the data. CR, AV, LMo, LMa, MC, VR, FM, and CD discussed data and wrote the manuscript. All authors contributed to the article and approved the submitted version.

## Funding

The study was not funded; no researcher received grants, salary, or reimbursements for the realization of the study. Human research ethical approval to conduct the survey was received from Florence University Ethics Committee (Prot. n. 0233044–21/12/2020).

## Conflict of interest

The authors declare that the research was conducted in the absence of any commercial or financial relationships that could be construed as a potential conflict of interest.

## Publisher’s note

All claims expressed in this article are solely those of the authors and do not necessarily represent those of their affiliated organizations, or those of the publisher, the editors and the reviewers. Any product that may be evaluated in this article, or claim that may be made by its manufacturer, is not guaranteed or endorsed by the publisher.

## Footnotes

^1^ www.surveymonkey.com
