# Supplementary material for: Post-traumatic stress symptoms and burnout in healthcare professionals working in neonatal intensive care units: Results from the STRONG study
Source: Front Psychiatry. 2023 Feb 3;14:1050236. doi: 10.3389/fpsyt.2023.1050236 (PMC9935564; doi:10.3389/fpsyt.2023.1050236)
Supplement: Supplementary file 1 [file Table_1.DOCX]

|  |  | **MBI** |  |  | **IES-R** |  |
| --- | --- | --- | --- | --- | --- | --- |
|  | Emotional Exhaustion | Reduced Personal Accomplishment | Depersonalisation | Total score | Intrusion | Avoidance |
| **MBI** |  |  |  |  |  |  |
| Reduced Personal Accomplishment | 0.199 | 1.000 |  |  |  |  |
| *p* | *0.0087* |  |  |  |  |  |
| Depersonalisation | 0.442 | 0.315 | 1.000 |  |  |  |
| *p* | *<0.0001* | *<0.0001* |  |  |  |  |
| **IES-R** |  |  |  |  |  |  |
| Total score | 0.410 | 0.261 | 0.265 | 1.000 |  |  |
| *p* | *<0.0001* | *0.0006* | *0.0005* |  |  |  |
| Intrusion | 0.308 | 0.244 | 0.158 | 0.915 | 1.000 |  |
| *p* | *0.0001* | *0.0015* | *0.0409* | *<0.0001* |  |  |
| Avoidance | 0.380 | 0.190 | 0.284 | 0.844 | 0.600 | 1.000 |
| *p* | *<0.0001* | *0.0139* | *0.0002* | *<0.0001* | *<0.0001* |  |
| Hyperarousal | 0.442 | 0.256 | 0.293 | 0.921 | 0.820 | 0.681 |
| *p* | *<0.0001* | *0.0009* | *0.0001* | *<0.0001* | *<0.0001* | *<0.0001* |

Supplementary Table 1: correlation coefficients (Pearson’s r) of all psychometric subscales.
